# **Equitable Access to Antibiotics:** A Core Element and Shared Global Responsibility for Pandemic Preparedness and Response

**DOI:** 10.1017/jme.2022.77

**Published:** 2022

**Authors:** Mengying Ren, Anthony D. So, Sujith J. Chandy, Mirfin Mpundu, Arturo Quizhpe Peralta, Kerstin Åkerfeldt, Anna Karin Sjöblom, Otto Cars

**Affiliations:** 1: UPPSALA UNIVERSITY, UPPSALA, SWEDEN; 2: JOHNS HOPKINS BLOOMBERG SCHOOL OF PUBLIC HEALTH, BALTIMORE, MD, USA; 3: CHRISTIAN MEDICAL COLLEGE, VELLORE, INDIA; 4: REACT AFRICA, LUSAKA, ZAMBIA; 5: REACT LATIN AMERICA, CUENCA, ECUADOR

**Keywords:** Antibiotics, Antibiotic Resistance, Pandemic Instrument, Equitable Access, Health System

## Abstract

Securing equitable antibiotic access as an essential component for health system resilience and pandemic preparedness requires a systems perspective. This article discusses key components that need to be coordinated and paired with adequate financing and resources to ensure antibiotic effectiveness as a global public good, which should be central while discussing a new global agreement.

## 1. Introduction

Access to effective antibiotics is the cornerstone of a functioning health system and a mainstay for responding to emerging health threats and achieving universal health care.^1^ Immediate and critical treatment of infectious diseases including hospital-associated infections are under threat due to the lack of access to effective antibiotics. Globally, the ongoing crisis of antibiotic resistance already claimed 1.27 million deaths in 2019, with a disproportionate burden on low- and middle-income countries (LMICs).^2^ 214,000 newborns are estimated to die every year from sepsis caused by resistant bacteria — representing at least 30% of all sepsis deaths in newborns.^3^It is concerning that lack of access to effective antibiotics causes more deaths than antibiotic resistance itself, though this may change with rising resistance.^4^

The responses sparked by COVID-19 led to unprecedented collaboration and action in rapid innovation of diagnostics, vaccines, and treatments. At the same time, the pandemic exposed systemic and deeply unacceptable global health inequalities. The intense discussions around equitable access to both innovations and basic supplies that are critical for pandemic preparedness and response sparked the process for developing a pandemic instrument.^5^ It is important that such an instrument is not limited to viral threats. Antibiotics are essential not only to manage current and forthcoming bacterial pandemics, but also to treat bacterial complications following viral infections and to manage many non-communicable diseases, such as cancer.

Global action to secure sustainable access to antibiotics has so far been too fragmented. In the context of a pandemic instrument, lessons from COVID-19 should increase the understanding of the need for addressing this critical issue from a systems perspective where essential functions — from prevention to access — are strengthened and coordinated. Establishing operating principles and mechanisms (with associated resource mobilization and allocations) to ensure global equitable access to effective antibiotics should be central in the discussion of a new global agreement.

## 2. Equitable Access to Effective Antibiotics

Over the past two decades, human antibiotic consumption globally has risen 46%, from 9.8 to 14.3 defined daily doses (DDD) per 1000 population per day.^6^ However, these figures mask the reality that many LMICs still have limited access to antibiotics.^7^ Essential to health systems, the antibiotic access issue is complex and rooted in scientific (e.g., research and development), financial (e.g., affordability) and structural (e.g., registration, distribution and appropriate use) barriers.^8^ Managing antibiotic effectiveness as a global public good ^9^ is not only related to the obstacles in bringing existing and novel antibiotics to patients in need, but also to challenges with awareness, attitudes, and practices on appropriate antibiotic use, as well as insufficient preventive measures, diagnostic capacity, and infrastructure.

Global action to secure sustainable access to antibiotics has so far been too fragmented. In the context of a pandemic instrument, lessons from COVID-19 should increase the understanding of the need for addressing this critical issue from a systems perspective where essential functions — from prevention, to access — are strengthened and coordinated. Establishing operating principles and mechanisms (with associated resource mobilization and allocations) to ensure global equitable access to effective antibiotics should be central in the discussion of a new global agreement.

To achieve equitable access to effective antibiotics, preventive measures and diagnostics are important elements not to be neglected. It is important to adopt a health systems approach for sustainable access to antibiotics, implying coordinated specific actions and concerted efforts towards health infrastructure, health workforce, and quality care. Appropriate preventive strategies can reduce infections and consequently the need for antibiotics. However, in many health systems, hygiene principles and the basics of infection prevention and control (IPC), including vaccination coverage, are less than optimal, and what is more concerning is that only 52% of health care facilities in sub-Saharan Africa had a basic water service in 2021. Globally, 1.7 billion people still lacked basic water service at their health care facility.^10^

Availability of point-of-care diagnostics could significantly improve treatment outcomes by providing information about which antibiotic to use and avoiding unnecessary or incorrect use, reducing total amount of antibiotic consumption and securing access in the long run. Although there have been recent scientific developments,^11^ new diagnostic tools are not globally available. Treatment must rely on laboratory-derived surveillance data, but the lack of quality-accredited laboratories, especially in rural areas in LMICs, poses a big challenge. The recent attempt for systematic monitoring of antibiotic resistance by the Global Antimicrobial Resistance Surveillance System (GLASS) has helped.^12^ However, GLASS data are often not used at the country level to guide practices or policies. Furthermore, the resistance data are skewed towards tertiary care hospitals in large cities that have microbiology laboratories. Point prevalence studies to supplement routine surveillance for guidance of treatment are urgently needed. Global integrated surveillance such as environmental surveillance (e.g., global wastewater surveillance) should be explored for rapid detection of resistance trends and early detection of novel resistance genes.

A reliable supply of essential antibiotics is critical to enable the use of the WHO Access, Watch, Reserve (AWaRe) classification,^13^ thus optimizing healthcare spending and reducing risks for resistance development. However, AWaRe implementation in LMICs has been limited, partly because of perennial stockouts, as well as supply chain and inventory control challenges.^14^ Many primary healthcare facilities do not even have ‘Access’ antibiotics, let alone ‘Watch’ antibiotics. Due to budgetary issues, many tertiary care hospitals have low inventory of ‘Reserve’ antibiotics. The challenges in access, however, are sometimes not seen in the private healthcare facilities in urban settings, but where issues of profitable sales and antibiotic excess instead often overpower issues of access.^15^

**Figure 1:**
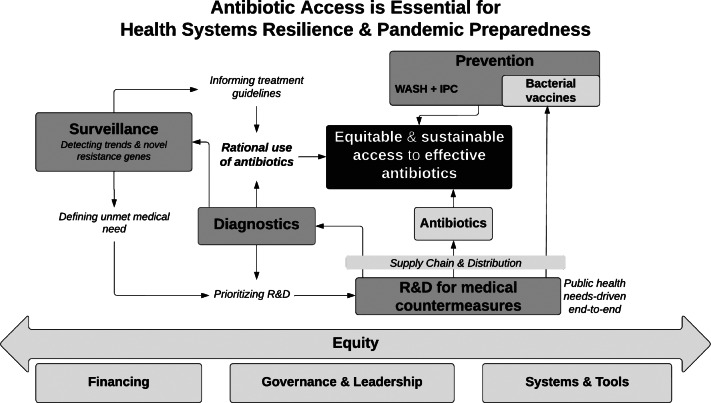
Key Components to Be Coordinated for Sustainable Access to Effective Antibiotics That Are Essential for Health Systems Resilience and Pandemic Preparedness

Figure 1 illustrates a health systems approach with key components to be coordinated to secure sustainable access to antibiotics. These components synergistically form important building blocks in a pandemic instrument.

## 3. Reforming Antibiotic Innovation: From Robust Investment in Early Discovery to Equitable and Sustainable Access

While the antibiotic pipeline is not addressing public health priorities, global and national discussions need to move away from ‘market fixing’ which often focuses on how to re-enlist those multinational pharmaceutical companies that have abandoned antibiotic development within the constraints of their traditional business model. Today it is the academic research groups and small- and medium-sized enterprises (SMEs), the majority having fewer than 50 employees, that constitute the greatest innovation force for antibiotic development.^16^ These efforts must be incentivized and funded, including through the critical guidance from pipeline coordinators or similar platforms such as Antibacterial Drug Development Engine (ENABLE-2),^17^ Combating Antibiotic Resistant Bacteria (CARB-X), and Global Antibiotic Research and Development Partnership (GARDP).^18^ Strong public leadership is needed to define a mission-driven, end-to-end vision for the development of and sustainable access to effective new antibiotics. New alternative and transformative models need to be explored, which by design and intent serve global health needs.^19^

Effective access to antibiotics relies on systems that ensure their innovation, production, stewardship, access, and distribution. A pandemic instrument can help shape the enabling environment by which these goals are met. As part of such a negotiated framework, the platform for preparing for ongoing and future health threats might put in place these building blocks of an end-to-end approach, making such medical countermeasures affordable and available to those in need. By taking such an approach, the pandemic instrument would realign how the public sector shapes R&D and how these life-saving products are distributed.

Applying a framework of sharing resources, risk and rewards (3Rs framework) is critical to improve the innovation ecosystem. This involves moving beyond bet-by-bet, drug-by-drug approaches.^20^ To share resources, risks and rewards, it would be important to pool building blocks of knowledge - from biospecimens and research tools to key platform technologies. The Pandemic Influenza Preparedness Framework shows how fair returns from public sector investments might be made. It laid out a benefit-sharing arrangement: in exchange for access to influenza virus samples, manufacturers made voluntary financial contributions and committed to set aside a share of the resulting vaccine doses to help address unmet needs.^21^

The 3Rs framework calls for sharing rewards as an important part of the solution, including through delinking the cost of investment in R&D from the price and volume of sales, while ensuring fair returns on the public investment made. Making de-linkage work requires aligning new incentives along the R&D chain to safeguard affordability and sustainable access. Several such approaches received consideration at the WHO Fair Pricing Forum in 2021.^22^ A pandemic instrument could also carry forward measures for greater transparency of markets for health products, as called for under a World Health Assembly resolution adopted in 2019.^23^ Public financing of products for pandemic preparedness and response, including antibiotics, should come with conditions for affordability and access. Product development partnerships have sought to arrange for such upfront access commitments in novel drugs.^24^

Ensuring sustainable access to health products could also involve making commitments through public sector or non-profit sector manufacturing or by leveraging strategic pooled procurement. Middle-income countries, where most of those living in extreme poverty reside, might particularly benefit from such concerted global efforts. For TB drugs, the Global Drug Facility provides a useful example, whereby demand forecasting as well as making financing more predictable has enabled this pooled procurement facility to secure multiple, quality-assured suppliers for the smaller markets of second-line TB medicines.^25^ The SECURE (Expanding Sustainable Access to Antibiotics) initiative being developed by GARDP and WHO, with support from UNICEF, and the Clinton Health Access Initiative (CHAI), is another model aiming to build a global consortium of organizations and countries to address access barriers to new and existing antibiotics in LMICs.^26^ A pandemic instrument could bolster efforts to apply these various strategic levers in concert as part of an end-to-end approach to bring novel antibiotics to market. Global public leadership is urgently needed to follow through the recommendations from the UN Interagency Coordination Group on Antimicrobial Resistance (IACG) to strengthen global coordination, investment and information sharing around antibiotic R&D.^27^

## 4. Recommendations

As antibiotic resistance and its death toll are rapidly rising, antibiotic use is increasing and the pipeline of new antibiotics is failing, it is evident that current governance structures are insufficient to secure access to effective antibiotics. This points to a clear need for more resilient health systems and stronger global governance to effectively prevent, prepare and respond to health crises, including antibiotic resistance. A pandemic instrument could help facilitate the revitalization of political momentum and resolve the lack of global collective action and the slow or insufficient implementation of existing declarations and commitments.

Antibiotic effectiveness needs to be seen as a global public good, and its preservation is a shared global responsibility. To strengthen global coordination and responsibility, we call for securing equitable and sustainable access to effective antibiotics and other countermeasures to be a key consideration in the upcoming pandemic instrument. Equity should be a central objective, and specific measures not only to expand access to antibiotics, but also balance their availability against misuse must be considered, ensuring access without excess. To fulfill this overarching goal, the following elements are critical:

- Prevention of infections is one of the most cost-effective measures to mitigate the spread of infections and resistance development. WHO is calling for better use of existing vaccines and the development of new vaccines to tackle antimicrobial resistance.^28^ Bacterial vaccines with sufficient population coverage would require equitable access to decrease disease burden and further prevent the spread of resistant strains worldwide. Minimum standards, target setting and associated resource allocation for IPC and Water, Sanitation and Hygiene (WASH) should be foundational elements in the pandemic instrument that synergistically links to efforts in managing antibiotic resistance. These efforts should be further reinforced by global goals including achieving quality health for all, for universal health coverage.
- Surveillance and early warning are essential for timely detecting disease outbreaks, identifying mutations, monitoring their developments and spread. Data on resistance patterns are key to inform treatment guidelines. With much focus on a global surveillance system for viruses with pandemic potential, it is strategic and critical to build a surveillance system that also covers bacteria and indeed all types of infectious diseases. A cornerstone to future pandemic preparedness will be to build on existing infrastructure to support an effective global, integrated surveillance system and should explore how environmental surveillance (e.g., global wastewater surveillance system) could be applied.
- Diagnostics are critical in providing quality care, improving rational use and avoiding unnecessary or incorrect use, and securing antibiotic access in the long run. In addition, diagnostics serve as a source of information for surveillance programs and making antibiograms. A global repository of biospecimens like that supported by the Pandemic Influenza Preparedness Framework should be explored not only for diagnostics, but for vaccines, with benefit-sharing provisions. The future instrument should ensure that development of and access to diagnostics are adaptable to different levels of local contexts and complemented by the strengthening of infrastructure and human resources.
- Lastly, antibiotic drug resistance is an ineluctable evolutionary process. This means that the pipeline of antibiotics must remain robust and meet public health needs. A global instrument should urge public leadership by institutionalizing global prioritization, coordination and long-term funding of R&D activities, such as enabling licensing for public and not-for-profit production as well as establishing pooled procurement mechanisms that support appropriate use. New and strengthened existing mechanisms need to remain independent from potential conflict of interest with the private sector, and guided by transparency and oversight of an end-to-end approach to R&D.

All the above require sustainable financing and resources backed up by systems strengthening including infrastructure, human resources, systems and tools for successful implementation. A pandemic instrument should introduce standards and procedures, paired with adequate funding and resources, and establish clear mechanisms to monitor for accountability on commitments made. By establishing rules-based governance — including transparency of data and global sharing of knowledge and technology as well as equitable allocation and distribution - the instrument could contribute significantly to health system resilience and ensuring that effective antibiotics are secured for all.

Summary Box Key Messages for Policymakers

- Antibiotic effectiveness needs to be seen as a global public good, and its preservation, a shared global responsibility. There is an urgent need for governments to take globally coordinated action to ensure equitable and sustainable access for those in need.
- Prevention of infections is one of the most cost-effective measures for reducing the need to use antibiotics and managing antibiotic resistance. Minimum standards and target setting for IPC and WASH (with associated resource allocations) should be foundational elements in the pandemic instrument and reinforced by global goals including achieving quality health for all.
- Pandemic preparedness depends on an effective global, integrated surveillance system and should explore how environmental surveillance (e.g., global wastewater surveillance system) could be applied. The future instrument should support increasing diagnostic capacity, laboratory infrastructure, educated personnel, strengthening national surveillance systems in LMICs, and developing integrated analysis of data across the human, animal and environment sector that account for both viral and bacterial threats.
- A pandemic instrument should introduce standards and procedures, and establish clear mechanisms for monitoring for accountability, paired with adequate financing and resources for implementation. Public leadership is needed to institutionalize global prioritization, coordination and long-term funding of R&D activities. By ensuring adequate resources for LMICs to follow up mandates and measures, and establishing rules-based governance – including transparency of data and global sharing of knowledge and technology as well as equitable allocation and distribution – the instrument could contribute significantly to health systems resilience and securing sustainable access to effective antibiotics for all.

## Note

The authors report grants from Sida, Swedish development aid agency during the conduct of the study. Dr. So also reports personal fees from Medecins sans Frontieres outside the submitted work; and service as a Member of the Board of Public Citizen, Inc. -- a non-profit consumer advocacy organization .

## References

[1] O. Cars , S.J. Chandy , M. Mpundu , A.Q. Peralta , A. Zorzet , and A.D. So , “Resetting the Agenda for Antibiotic Resistance through a Health Systems Perspective,” The Lancet Global Health 9, no. 7 (2021): e1022–e1027. 34143980 10.1016/S2214-109X(21)00163-7 PMC9237786

[2] C.J. Murray , K.S. Ikuta , F. Sharara , et al., “Global Burden of Bacterial Antimicrobial Resistance in 2019: A Systematic Analysis,” The Lancet 399, no. 10325 (2022): 629–655. 10.1016/S0140-6736(21)02724-0 PMC8841637 35065702

[3] S.C.H. Wen , Y. Ezure , L. Rolley , et al., “Gram-Negative Neonatal Sepsis in Low- and Lower-Middle-Income Countries and WHO Empirical Antibiotic Recommendations: A Systematic Review and Meta-analysis,” in Z.A. Bhutta , ed., PLOS Medicine 18, no. 9 (2021): e1003787. 34582466 10.1371/journal.pmed.1003787 PMC8478175

[4] N. Daulaire , A. Bang , G. Tomson , J.N. Kalyango , and O. Cars , “Universal Access to Effective Antibiotics Is Essential for Tackling Antibiotic Resistance,” Journal of Law, Medicine & Ethics 43, no. 3, Suppl. (2015): 17–21. 10.1111/jlme.12269 26243238

[5] *World Health Assembly Agrees to Launch Process to Develop Historic Global Accord on Pandemic Prevention, Preparedness and Response*, News Release, December 1, 2021, *available at* <https://www.who.int/news/item/01-12-2021-world-health-assembly-agrees-to-launch-process-to-develop-historic-global-accord-on-pandemic-prevention-preparedness-and-response> (last visited September 20, 2022).

[6] A.J. Browne , M.G. Chipeta , G. Haines-Woodhouse , et al., “Global Antibiotic Consumption and Usage in Humans, 2000-18: A Spatial Modelling Study,” The Lancet Planet Health 5, no. 12 (2021): e893–e904. 34774223 10.1016/S2542-5196(21)00280-1 PMC8654683

[7] C. Kållberg , C. Årdal , H. Salvesen Blix , et al., “Introduction and Geographic Availability of New Antibiotics Approved between 1999 and 2014,” PLOS ONE 13, no. 10 (2018): e0205166. 30325963 10.1371/journal.pone.0205166 PMC6191083

[8] A.D. So , M. Bigdeli , G. Tomson , W. Woodhouse , E. Ombaka , A.Q. Peralta , “Part 5: The Access and Excess Dilemma,” in “Antibiotic Resistance: The Need for Global Solutions,” The Lancet Infectious Diseases 13, no. 12 (2013): 1057–1098. 24252483 10.1016/S1473-3099(13)70318-9

[9] S. Moon , J.A. Røttingen , and J. Frenk , “Global Public Goods for Health: Weaknesses and Opportunities in the Global Health System,” Health Economics, Policy and Law 12, no. 2 (2017): 195–205. 28332461 10.1017/S1744133116000451

[10] “Progress on WASH in Health Care Facilities 2000-2021: Special Focus on WASH and Infection Prevention and Control (IPC),” WHO/UNICEF Joint Monitoring Programme (JMP) for Water Supply, Sanitation and Hygiene (WASH) August 2022, *available at* <https://data.unicef.org/resources/jmp-wash-in-health-care-facilities-2022/> (last visited Dec. 6, 2022).

[11] R.K. Shanmugakani , B. Srinivasan , M.J. Glesby , et al., “Current State of the Art in Rapid Diagnostics for Antimicrobial Resistance,” Lab Chip 20, no. 15 (2020): 2607–2625. 32644060 10.1039/d0lc00034e PMC7428068

[12] WHO, “Global Antimicrobial Resistance and Use Surveillance System (GLASS),” *available at* <https://www.who.int/initiatives/glass> (last visited September 20, 2022).

[13] WHO, “2021 AWaRe classification,” *available at* <https://www.who.int/publications/i/item/2021-aware-classification> (last visited September 20, 2022).

[14] E. Lugada , H. Komakech , I. Ochola , S. Mwebaze , M. Olowo Oteba , and D. Okidi Ladwar , “Health Supply Chain System in Uganda: Current Issues, Structure, Performance, and Implications for Systems Strengthening,” Journal of Pharmaceutical Policy and Practice 15, no. 1 (2022): 14; K. Loosli, A. Davis, A. Muwonge, and T. Lembo, “Addressing Antimicrobial Resistance by Improving Access and Quality of Care: A Review of the Literature from East Africa,” *PLoS Neglected Tropical Diseases* 15, no. 7 (2021): e0009529. 34292932 10.1371/journal.pntd.0009529 PMC8297743

[15] S. KIK , S.J. Chandy , L. Jeyaseelan , R. Kumar , S. Suresh , “Antimicrobial Prescription Patterns for Common Acute Infections in Some Rural & Urban Health Facilities of India,” Indian Journal of Medical Research 128, no. 2 (2008): 165–171. 19001680

[16] WHO, “Antibacterial Agents in Clinical and Preclinical Development: An Overview and Analysis,” May 27, 2022, *available at* <https://www.who.int/publications/i/item/9789240047655> (last visited September 20, 2022).

[17] ENABLE-2 is an antibacterial drug discovery platform based on learnings from the European Gram-negative Antibacterial Engine (ENABLE) within the EU Innovative Medicines Initiative, *available at* <https://www.ilk.uu.se/enable2/> (last visited Dec. 6, 2022).

[18] E. Baraldi, F. Ciabuschi, S. Callegari, and O. Lindahl, “*Economic Incentives for the Development of New Antibiotics: Report Commissioned by the Public Health Agency of Sweden*,” Uppsala Universitet, 2019, *available at* <http://urn.kb.se/resolve?urn=urn:nbn:se:uu:diva-375258> (last visited September 20, 2022).

[19] ReAct Report: Governments Need to Take More Leadership to Ensure Global Sustainable Access to Effective Antibiotics,” 2021, *available at* <https://www.reactgroup.org/news-and-views/news-and-opinions/year-2021/new-react-report-governments-need-to-take-more-leadership-to-ensure-global-sustainable-access-to-effective-antibiotics/> (last visited September 20, 2022).

[20] A.D. So , Q. Ruiz-Esparza , N. Gupta , and O. Cars , “3Rs for Innovating Novel Antibiotics: Sharing Resources, Risks, and Rewards,” BMJ 344 (2012): e1782. 22491076 10.1136/bmj.e1782

[21] WHO, “PIP@10 — Celebrating a Decade of Implementation,” March 8, 2022*, available at* <https://www.who.int/publications/m/item/pipat10---celebrating-a-decade-of-implementation> (last visited September 20, 2022).

[22] WHO, *Fair Pricing Forum 2021: Forum Discussion Paper: Aligning Incentives for Pharmaceutical Innovation to Achieve Fair Pricing*, 2021, *available at* <https://apps.who.int/iris/handle/10665/348289> (last visited September 20, 2022).

[23] World Health Assembly 72, *Improving the Transparency of Markets for Medicines, Vaccines, and Other Health Products (FOOTNOTE): Draft Resolution Proposed by Andorra, Brazil, Egypt, Eswatini, Greece, India, Italy, Kenya, Luxembourg, Malaysia, Malta, Portugal, Russian Federation, Serbia, Slovenia, South Africa, Spain, Sri Lanka, Uganda*, World Health Organization, 2019, *available at* <https://apps.who.int/iris/handle/10665/329172> (last visited September 20, 2022).

[24] GARDP, *GARDP and Entasis Therapeutics Initiate Global Phase 3 Trial of Zoliflodacin, a First-in-Class Oral Antibiotic for the Treatment of Gonorrhoea*, Press Release, September 30, 2019, *available at* <https://gardp.org/news-resources/gardp-and-entasis-therapeutics-initiate-global-phase-3-trial-of-zoliflodacin-a-first-in-class-oral-antibiotic-for-the-treatment-of-gonorrhoea/> (last visited September 20, 2022); GARDP, *Shionogi, GARDP and CHAI Announce Landmark License and Collaboration Agreements to Treat Bacterial Infections by Expanding Access to Cefiderocol in 135 Countries*, Press Release, June 15, 2022, *available at* <https://gardp.org/news-resources/shionogi-gardp-and-chai-announce-landmark-license-and-collaboration-agreements/> (last visited September 20, 2022).

[25] N. Arinaminpathy , T. Cordier-Lassalle , K. Lunte , and C. Dye , “The Global Drug Facility as an Intervention in the Market for Tuberculosis Drugs,” Bulletin of the World Health Organization 93, no. 4 (2015): 237–248A. 26229188 10.2471/BLT.14.147256 PMC4431561

[26] WHO, “Expanding Sustainable Access to Antibiotics (SECURE),” *available at* <https://www.who.int/groups/secure-expanding-sustainable-access-to-antibiotics> (last visited September 20, 2022).

[27] UN Interagency Coordination Group (IACG) on Antimicrobial Resistance, “No Time to Wait: Securing the Future from Drug-Resistant Infections,” April 19, 2019, *available at* <https://www.who.int/publications/i/item/no-time-to-wait-securing-the-future-from-drug-resistant-infections> (last visited September 20, 2022).

[28] WHO, *Urgent Call for Better Use of Existing Vaccines and Development of New Vaccines to Tackle AMR*, July 12, 2022, *available at* <https://www.who.int/news/item/12-07-2022-urgent-call-for-better-use-of-existing-vaccines-and-development-of-new-vaccines-to-tackle-amr> (last visited September 20, 2022). PMC9749679 35964964

